# Exposure to secondhand smoke among school-going adolescents in Malaysia: Results from the National Health and Morbidity Survey (NHMS) 2022: Adolescent Health Survey (AHS)

**DOI:** 10.18332/tid/192002

**Published:** 2024-10-25

**Authors:** Kuang Hock Lim, Yoon Ling Cheong, Kuang Kuay Lim, Mohd Ruhaizie Riyadzi, Hamizatul Akmal Abd Hamid, Jia Hui Lim, Chee Cheong Kee, Sumarni Mohd Ghazali, Hui Li Lim

**Affiliations:** 1 Institute for Medical Research, National Institutes of Health, Ministry of Health Malaysia, Shah Alam, Malaysia; 2 Institute for Public Health, National Institutes of Health, Ministry of Health Malaysia, Shah Alam, Malaysia; 3 Faculty of Allied Science, Universiti Kebangsaan Malaysia, Bangi, Malaysia; 4 Biostatistics and Data Repository Sector, National Institutes of Health, Shah Alam, Malaysia; 5 Institute for Clinical Research, National Institutes of Health, Ministry of Health Malaysia, Shah Alam, Malaysia

**Keywords:** secondhand smoke, school-going adolescents, displacement of smoking, NHMS 2022, AHS

## Abstract

**INTRODUCTION:**

The adverse effects of secondhand smoke (SHS) exposure on health have been well established. Using the NHMS 2002: Adolescent Health Survey (AHS), this study attempts to evaluate the extent and factors associated with SHS exposure among Malaysian school-going adolescents.

**METHODS:**

We conducted the NHMS 2022: AHS to gather a representative sample of school-age teenagers. We employed a cross-sectional study design and a multi-stage sampling procedure. We utilized a pre-validated self-administered questionnaire to collect data from the selected respondents. The data analysis included adjusted odds ratios (AORs) with 95% confidence intervals (95% CIs). Additionally, we investigated the possible two-way interactions between the independent variables.

**RESULTS:**

The SHS prevalence was 42.0%. Exposure to secondhand smoke (SHS) was associated with male gender (AOR=1.28; 95 CI: 1.20–1.38), older age (AOR=1.46; 95% CI: 1.33–1.60), Malay (AOR=1.88; 95% CI: 1.49–2.37), Bumiputra Sabah (AOR=2.23; 95% CI: 1.67–2.99), Bumiputra Sarawak (AOR=2.43; 95% CI: 1.80–3.28), and Chinese ethnicity (AOR=2.89; 95 CI: % 2.30–3.64), as well as current smoking (AOR=2.78; 95% CI: 2.50–3.09), having separated or divorced parents (AOR=1.12; 95% CI: 1.02–1.23), and parental tobacco product use (AOR=4.75; 95% CI: 4.44–5.08). We found significant interactions between: 1) Age group with gender and ethnicity; 2) Gender and ethnicity; and 3) Parental smoking status with gender, response to tobacco use, parents' marital status, and ethnicity.

**CONCLUSIONS:**

Parental characteristics, sociodemographic characteristics, and SHS exposure are strongly correlated. In addition, there is evidence of smoking displacement to the house from other areas by parents/guardians. This study offers a fresh perspective on how these variables influence the likelihood of SHS exposure for Malaysian school-age teenagers. More efforts should focus on parental variables and sociodemographic traits, especially parental smoking cessation support.

## INTRODUCTION

Exhaled mainstream smoke (the smoke exhaled by the smoker) and sidestream smoke (the smoke produced from the burning end of a cigarette) make up secondhand smoke (SHS)^1^. These substances include more than 200 known respiratory toxins and carcinogens, such as formaldehyde, benzene, 1,3-butadiene, hydrogen cyanide, and mercury^2^. One’s health could be impacted by SHS exposure. Diseases related to SHS were among the leading risk factors for deaths globally in 2019, accounting for approximately 1.3 million deaths and contributing to 37 million disability-adjusted life years (DALYs), with 11.2% of the burden in children aged <5 years^3^. Exposure to SHS has been demonstrated to impede young people’s neurodevelopment and is strongly linked to low academic success and neurocognitive function in young people^4,5^. In addition, it is related to lower respiratory conditions such as middle ear disease in children, dyspnea in school-age children, low birth weight, history of asthma, and reduced lung function in childhood^4^.

Additionally, SHS has been linked to a higher risk of atopy-immunoglobulin E-mediated allergy^4^, obstructive sleep apnea (OSA) in children, single or multiple allergic morbidity^6^, sleep quality, and depressive symptoms^5-7^. In addition to its adverse impacts on health, SHS exposure makes non-smokers more susceptible to smoking and lowers the chance that young smokers would give up their habit^8^. Consequently, one of the goals listed in Malaysia’s national strategy plan for tobacco control is to lower SHS exposure^9^. This is in line with the rectification of the Framework Convention on Tobacco Control (FCTC) by the World Health Organization^10^ by the Malaysian government, ratified in 2005; the Control of Tobacco Products Regulations (CTPR) 2004^11^ had been amended several times to establish non-smoking areas in public places. Several public spaces have been designated as non-smoking zones, including restaurants with or without air conditioning, government offices, offices, healthcare facilities, public transport, schools, cultural institutions, indoor stadiums, etc. Additionally, the Ministry of Health, Malaysia, and state governments have collaborated to implement the idea of non-smoking zones in specific places in the state. For example, Melaka (Melaka Bebas Asap Rokok, MBAR)^12^. In addition, the Ministry of Health Malaysia and the Ministry of Education collaborate to promote health and other anti-smoking school activities.

In line with the ratification of the FCTC by the Malaysian government, various studies have been carried out to meet the requirements of the FCTC measure^13,14^, among which are periodic studies at the national level, such as the National Morbidity and Morbidity Survey (NHMS) conducted every four years from 2011 to 2015, and the Malaysia Global School Health Survey conducted among secondary school-going students once every five years. The M-GSHS 2012 (41.6%) and AHS survey 2017 (42.4%) reported that 4 out of 10 respondents had been exposed to SHS in the past week^13^. The study found no change in the prevalence and factors associated with exposure to SHS among males, older adolescents (aged ≥16 years), Malays, and other Bumiputras who report high odds of exposure to SHS in Malaysia^13^.

In line with the recommendation of the World Health Organization Framework Convention on Tobacco Control (FCTC) to use the MPOWER approach (monitoring tobacco use, protecting people from tobacco smoke, quitting smoking, and warning about the danger of tobacco) measures for effective interventions to reduce the demand for tobacco and restrict tobacco use, this article aims to examine the current prevalence and factors related to SHS among secondary school adolescents in Malaysia, using data from the adolescent NHMS 2022: AHS^15^, which was carried out at the national level.

## METHODS

We conducted the nationwide NHMS 2022: AHS survey from June to July 2022. The cross-sectional study design and the multi-stage sample were employed to select a representative sample of secondary school adolescents using the sampling frame of 2021 provided by the Ministry of Education, encompassing private and public secondary schools under the Ministry of Rural and Regional Development (MARA) secondary school in Malaysia The first phase in the sampling method is the stratification of each state in Malaysia followed by stratification of each state into the urban and rural areas. The primary sampling units (schools) in each state were selected using proportionate-to-size sampling, and three to nine classes were randomly selected from each school using systematic random sampling. Every student in the classes chosen received an invitation to participate in the study. The Ministry of Education, Malaysia and state and District education departments approved the study’s protocol.

Furthermore, the Medical Research Ethics Committee of the Ministry of Health Malaysia granted ethical approval for the study. This study’s sample size is based on each module’s objectives using a single proportion formula based on the prevalence of the previous adolescent health survey (NHMS 2017) conditions, the largest sample size used. The design effect of two and non-response rates of 20% are considered. Thus, 36000 adolescents were required for national-level analysis and 2250 adolescents for state-level analysis.

The active consent strategy was employed in the surveys to obtain consent from the respondents’ parents or guardians. The family received the consent form and a letter from the school administration. The letter detailed the purpose of the study and the guidelines for participating in it. The completed form was returned to the school administration by parents or guardians who gave their permission.

Data were obtained from the selected respondents by trained research team members who performed a briefing session of the respondents before data collection, outlining the survey’s objectives, the questionnaire’s contents, their freedom to decline to respond to any question, and the confidentiality of the information provided. Respondents needed to complete a consent form in the survey session. Research team members assisted the respondents who needed help to understand the items in the questionnaire or had any queries about the questionnaire’s content.

The following item was used to quantify the dependent variable (exposure to SHS): ‘Over the last seven days, how many days did people smoke in your presence?’. Pupils who gave the response ‘0 days’ were classified as ‘Not exposed to SHS’, but those who gave the responses: 1–2 days, 3–4 days, 5–6 days, or all seven days were classified as exposed to SHS16. The independent variables were: gender, age (13–15 years or ≥16 years), current smokers (if they used any smoked tobacco products in the past 30 days, including manufactured cigarettes, roll-your-own cigarettes, traditional hand-rolled cigarettes, shisha, cigar, or pipe), parental smoking status (non-smoking, or at least one parent smoked), marital status of the parents (married or separated/divorced) and ethnicity (Malay, Chinese, Indian, Bumiputra Sabah, Bumiputra Sarawak, and other).

### Data management and analysis

Data were cleaned two times before analysis to ensure the quality of the data, first by a research team member in the field and then by the research management team at the institution level. In addition, we randomly selected 10% of the data and compared it with the hard copy data to ensure the consistency of the data obtained. Furthermore, we ran the frequency analysis for each variable to detect any missing or outlier values in the data. Data were weighted based on the change of respondents to be selected (there is the inverse of the probability of choosing each school times the inverse of the probability of selecting each classroom times a school-level non-response adjustment factor times a student-level non-response adjustment factor calculated by classroom). We used descriptive statistics to describe the sociodemographic characteristics of the participants. Chi-squared analysis was employed to determine the association between the exposure and the dependent variable (exposure to SHS). A multivariable logistic regression model containing all the independent variables that achieved a p≤0.25 in bivariate analyses was fitted. We calculated the AOR and 95% CI for the main effect. In addition, the resulting model was evaluated for every conceivable two-way (multiplicative) interaction between the independent variables. The average predictive probability values were obtained by computing the predicted probabilities for SHS exposure based on the final model fitted with significant interaction terms. We also assessed the value of the log-likelihood change between the main effect and the main effect with an interaction effect. We used the SPSS statistics program version 2018 complex samples module to perform all statistical analyses (two-tail) at 95% significance.

## RESULTS

The total of 33523/37478 responses to the study gave a response rate of 89.4% (n=33523). The proportion of gender was equally distributed (male 50%), with more than 6 in 10 respondents aged 13–15 years and of Malay ethnicity. More than 40% of the respondents had at least one parent/guardian who smoked, and almost 20% of the respondents were current tobacco users (Table 1).

**Table 1:** Sociodemographic characteristics of school-going adolescents who participated in the National Health and Morbidity Survey 2022: Adolescent Health Survey (NHMS 2022: AHS) (N=33523)

*Characteristics*	*Estimated population*	*Sample*	*%*
**Gender**			
Male	1038709	15493	50.0
Female	1038386	18030	50.0
**Age** (years)			
13–15	1300006	20535	62.6
≥16	777090	12988	37.4
**Ethnicity**			
Malay	1307669	23125	63.0
Chinese	376349	5085	18.1
Indian	123746	1556	6.0
Bumiputra Sabah	117014	1722	5.6
Bumiputra Sarawak	105623	1241	5.1
Other	46393	794	2.2
**Parental smoking**			
None	1106348	17956	56.6
At least one	847724	13642	43.4
**Smoking status**			
Non-smokers	1686278	27511	81.5
Current smokers	381747	5869	18.5
**Parental marital status**			
Married	1734157	28070	85.1
Divorce/separated	302907	4844	14.9

Table 2 shows that 41.5% of respondents were exposed to SHS during the last seven days. A significantly higher proportion of exposure was observed among current tobacco users (65.6% vs 36.5; 1.78 times higher) and those with at least one parent who smoked (62.6 vs 26.0%; 2.4 times higher). Similarly, Males, Malay, Chinese, Bumiputra Sabah, and Sarawak ethnicities had two times higher exposure than respondents of Indian ethnicity.

**Table 2:** Exposure to secondhand smoke among school-going adolescents by sociodemographic variables, parental smoking status, and smoking status among participants in the National Health and Morbidity Survey 2022: Adolescent Health Survey (NHMS 2022: AHS) (N=33523)

*Variables*	*Exposure to SHS*				*χ^2^ (df)*	*p*
	*Yes*		*No*			
	*n*	*% (95% CI)*	*n*	*% (95% CI)*
**Gender**						
Male	6870	45.7 (43.9–47.5)	8516	54.3 (525–56.1)	212.15 (1)	<0.001
Female	6524	37.8 (36.5–39.2)	11432	62.2 (60.8–63.5)		
**Age** (years)						
13–15	7397	38.1 (36.7–39.6)	12994	61.9 (60.4–63.3)	300.94 (1)	<0.001
≥16	5997	47.8 (45.9–49.7)	6954	52.2 (50.3–54.1)		
**Ethnicity**						
Malay	9229	42.3 (40.6–44.0)	13779	57.7 (56.0–59.4)	420.21 (4.01)	<0.001
Chinese	2151	43.3 (40.4–46.2)	2910	56.7 (53.8–59.6)		
Indian	318	20.7 (17.7–24.0)	1226	79.3 (76.0–82.7)		
Bumiputra Sabah	759	45.1 (39.4–51.0)	950	54.9 (49.0–60.6)		
Bumiputra Sarawak	591	49.2 (45.1–53.3)	648	50.8 (46.7–54.9)		
Other	346	45.2 (40.4–50.2)	435	54.8 (49.8–59.6)		
**Parental smoking**						
None	4347	26.0 (24.8–27.2)	13603	74.0 (72.8–75.2)	4269.82 (1)	<0.001
At least one	8354	62.6 (61.0–64.0)	5281	37.4 (36.0–39.0)		
**Smoking status**						
Non-smokers	9647	36.5 (35.2–37.7)	17835	63.5 (62.3–64.8)	1708.5 (1)	<0.001
Current smokers	3746	65.2 (63.1–67.3)	2113	34.8 (32.7–36.9)		
**Parental marital status**						
Married	11074	41.3 (40.0–42.6)	16853	58.7 (57.4–60.0)	30.46 (1)	0.001
Divorce/separated	2104	45.5 (43.4–47.6)	2711	54.5 (52.4–56.6)		

Multivariable analysis was used to obtain odds of SHS exposure (Table 3). Significantly higher number of adolescents were exposed to SHS who had at least a parent smoker (AOR=4.75; 95% CI: 4.44–5.08), were current tobacco users (AOR=2.78; 95% CI: 2.50–3.99), were aged ≥16 years (AOR=1.46; 95% CI: 1.33–1.60), of Malay (AOR=1.88, 95% CI: 1.49–2.37), Chinese (AOR=2.89; 95% CI: 2.30–2.64), Bumiputra Sabah (AOR=2.23; 95% CI: 1.67–2.99), Bumiputra Sarawak (AOR=2.43; 95% CI: 1.80–3.28) and other ethnicity (AOR=2.08; 95% CI: 1.51–2.87) (with Indian ethnicity as reference). In addition, the log-likelihood value significantly increased from the primary effect model of 2077.75 to 6383.0 (main effect and interaction term between the independent variables).

**Table 3:** Multivariable logistic regression analysis of secondhand smoke exposure among school-going adolescents, by sociodemographic variables, parental smoking status, and smoking status among participants in the National Health and Morbidity Survey 2022: Adolescent Health Survey (NHMS 2022: AHS) (N=33523)

*Variables*	*AOR (95% CI)*	*p*
**Gender**		
Male	1.28 (1.20–1.38)	<0.001
Female ®	1	
**Age** (years)		
13–15 ®	1	
≥ 16	1.46 (1.33–1.60)	<0.001
**Ethnicity**		
Malay	1.88 (1.49–2.37)	<0.001
Chinese	2.89 (2.30–3.64)	
Indian ®	1	
Bumiputra Sabah	2.23 (1.67–2.99)	
Bumiputra Sarawak	2.43 (1.80–3.28)	
Other	2.08 (1.51–2.87)	
**Parental smoking**		
None ®	1	<0.001
At least one	4.75 (4.44–5.08)	
**Smoking status**		
Non-smokers ®	1	<0.001
Current smokers	2.78 (2.50–3.09)	
**Parental marital status**		
Married ®	1	0.021
Divorce/separated	1.12 (1.02–1.23)	

AOR: adjusted odds ratio.

® Reference categories.

**Figure 1:**
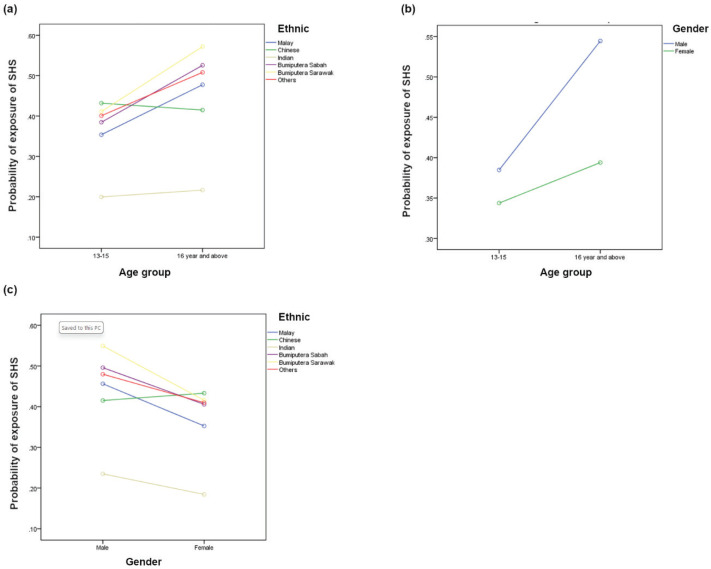
The interaction effect of age group and ethnicity (a), age group and gender (b), gender and ethnicity (c), among school-going adolescents who participated in the National Health and Morbidity Survey 2022: Adolescent Health Survey (NHMS 2022: AHS)

**Figure 2:**
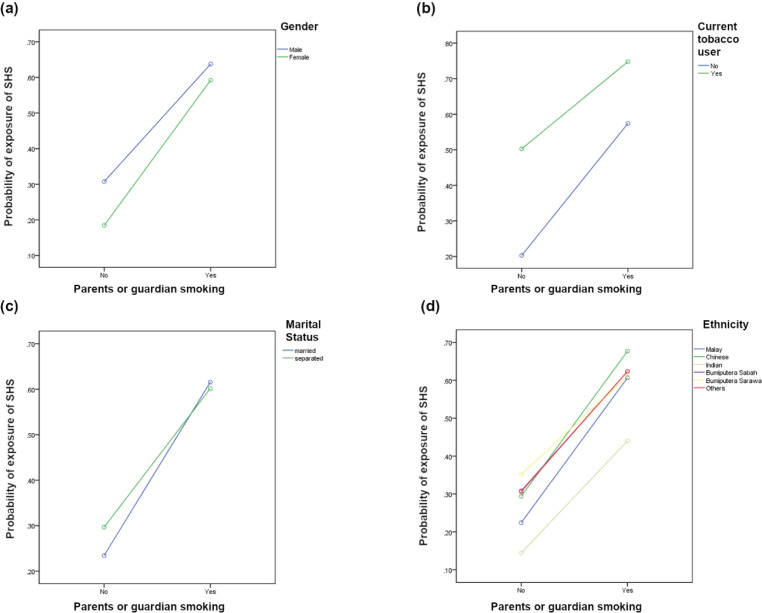
The interaction effect of parents or guardians smoking with gender (a), parents or guardians smoking with current tobacco users (b), parents or guardians with parental smoking status (c), and parents or guardians smoking with ethnicity (d), among school-going adolescents participated in the National Health and Morbidity Survey 2022: Adolescent Health Survey (NHMS 2022: AHS)

Significant interactions were found between: 1) age group, including age, ethnicity, gender, and current use of alcohol and drugs; 2) gender and ethnicity; and 3) parental smoking with gender, smoking status, parents’ marital status, and ethnicity (Figures 1 and 2). Supplementary file Table 1 depicts the probability of current e-cigarette use by examining interactions between various sociodemographic characteristics, lifestyle risk behaviors, and parental factors.

Figure 2d shows the parental smoking and ethnicity interaction, respondents with parent or guardian who smoked showed a higher probability of SHS exposure. The interaction also shows that differences in SHS exposure more significant effect among the high-risk group [respondents with at least one parental smoker (almost 30% to 38% difference)] in all ethnic groups than the low-risk group [respondents without parental smoker (10–23%)]. A parental smoker and marital status show cross-over interaction. Respondents with parents who smoked had a higher probability of SHS exposure (Figure 2c). The marital status of the parents had a more significant effect on respondents from SHS exposure in the low-risk group [respondents with no parents smokers (2.2% difference)] than the high-risk group [respondents with parent smokers (5.8 % difference)].

Figure 1 shows the: 1) interaction between age-group and sex, ≥16 years (male=54.9%, female=40.9%), 13–15 years (male=40.3%, female=36.0%); 2) interaction between age group and ethnicity, ≥16 years (male=51.1%, Chinese=40.9%, Indian=20.7%, Bumiputra Sabah=51.0%, Bumiputra Sarawak=57.2%, other=51.2%), 13–15 years (Malay=37.2 %, Chinese=44.8%, Indian=20.7%, Bumiputra Sabah=40.7%, Bumiputra Sarawak=44.1%, other=41.8%); and 3) interaction between gender and ethnicity group, male (Malay=47.4%, Chinese=42.1%, Indian=23.0%, Bumiputra Sabah=50.4%, Bumiputra Sarawak=55.8%, other=50.0%), female (Malay=37.1%, Chinese=44.5%, Indian=18.6%, Bumiputra Sabah=40.8%, Bumiputra Sarawak=42.3%, other=40.3%).

The gender-ethnicity interaction showed a cross-over interaction, with Chinese females (low-risk group) showing a higher probability of SHS exposure (2.4% difference) in contrast to other ethnic groups (Figure 1c), in which all female respondents of other ethnicities (e.g. Malay) showed lower exposure compared to male respondents. The analysis revealed the most significant difference in SHS exposure among the higher-risk group (males) and low-risk group (females), at 13.6% among Bumiputra Sarawak ethnicity.

Figure 2 show the: 1) interaction between parent/guardian smoking with gender, one or both parents (male=64.7%, female=60.6%), none (male=32.0%, female=19.7%); 2) interaction between parent/guardian smoking with status of tobacco user, one or both parents (yes=75.9%, no=58.4%), none (yes=51.2%, no=21.9%); 3) interaction between parent/guardian smoking with ethnicity, one or both parents (Malay=62.0%, Chinese=68.3%, Indian=47.9%, Bumiputra Sabah=61.8%, Bumiputra Sarawak=62.2%, other=64.7%), no parent/guardian smoking with ethnicity (Malay=24.5%, Chinese=30.4%, Indian=13.8%, Bumiputra Sabah=31.6%, Bumiputra Sarawak=36.9%, other=29.8%); and 4) interaction between parent/guardian smoking with parent marital status, parent/guardian smoking by one or both parents (married=62.7%, divorced/separated=62.5%), none (married=25.3%, divorced/separated=31.1%). Figure 2a shows that respondents with parents who smoked (higher risk group) had a higher probability of SHS compared to the low-risk group (respondents whose parents are not smokers) (12.3% vs 4.1%). Figure 2c also shows that current tobacco use had a greater probability of SHS exposure. The effect of SHS exposure use was more significant in the low-risk group [respondents with no parental smoker (29.3% difference)] than the high-risk group [at least one parent/guardian smoked (17.5% difference)].

## DISCUSSION

The study revealed that SHS exposure in 2022 was 41.5%, which is not significantly different from the prevalence in 2017 (42.0%) and 2012 (41.5%)^13^. In addition, the exact prevalence rate was reported by Ghazali et al.^17^ among youth in Malaysia (41.5%). However, this prevalence is lower than global youth exposure (62.9%) and for South-East Asia (65.3%)^18^. It is also lower than the reported 62.8% among girls in China^16^. In addition, it is also lower than Thailand’s youth exposure of 58.2%^7^. Similarly, Xi et al.^19^ have reported a prevalence of 55.9% in 68 low- and middle-income countries (LMICs). However, the no prevalence change since the last decade is disappointing, considering that various anti-smoking measures, such as community intervention programs such as KOSPEN and schools’ health promotion on the hazards of cigarettes etc., have been implemented from 2012 to 2022 in Malaysia. This finding contradicts the results of the study of Kuwabara et al.^20^ which showed a decrease of 14.5% in any public place and a 13.4% decrease in exposure to SHS at home for one day in the past seven days during the years 2008–2017. In addition, this finding also contradicts the findings of the study by Kuntz and Lampert^21^, which showed that within only six years, the proportion of non-smoking adolescents spending several times a week or even daily time in rooms where others are smoking decreased from 35.1% to 18.8%. Distinct reductions in smoking prevalence and SHS exposure could be seen in all SES groups^21^. The findings in this study are the same as those of the Lim et al.^13^ study which involved a comparison of SHS between 2012 and 2017, and the Gentzke et al.^22^ study among middle and high school students in the United States. The findings may be due to no change in the prevalence of smoking among adults and teenagers in several national-level studies conducted over the past decade, which causes no change in the prevalence rate of SHS^13,23^. The finding might also be due to the lack of awareness of the harmful effects of secondhand smoke among parents and adolescents, who are therefore less keen to avoid SHS exposure, thus explaining their higher likelihood of SHS exposure.

A multivariate analysis showed that several factors are significantly associated with exposure to SHS among school-going adolescents, including being male, smokers, with at least one parent smoking, being older adolescents (aged 16–18 years), and adolescents from divorced/separated families. These factors are almost identical to those in the M-GSHS 2012 and AHS 2017 studies^13^. The higher likelihood of smokers being more susceptible to SHS exposure may be due to adolescent smokers smoking together with their peers, as reported by Lim et al.^24^ in their study on smoking practices among high school students in Kota Tinggi district, where the results of their research show that almost 90% of teenage smokers smoke with friends. Lagerweij et al.^25^ and Kuwabara et al.^20^ also reported the same findings among adolescent smokers in Europe and Japan, respectively. This finding may be explained by human Behavioral Theory, which postulates that youth are likelier to befriend similar peers^26^. Adolescents with at least one parent who smoked showed higher prevalence and odds of a parent with SHS exposure; this is in line with the finding by Xi et al.^19^ among adolescents aged 12–15 years in 68 low- and middle-income countries. Ghazali et al.^17^ and Lim et al.^27^ also reported similar findings in their study in Malaysia.

People are more likely to smoke at home because the number of public places where smoking is permitted has shrunk as a result of Malaysia’s expanded smoking ban zones as a result of the Control of Tobacco Product Regulation amendments in 2004. Older adolescents have higher odds of being exposed to SHS compared to their counterparts who are aged 13–15 years; this may be explained by plausible factors, among which teenagers aged 16–18 years are more mobile since parents and guardians give more freedom to older adolescents compared to younger adolescents. This freedom makes them more likely to visit public places and increases the likelihood of exposure to SHS^13,17^. In addition, this observation may be explained by the higher prevalence of smoking among adolescents from this age group, given that respondents tend to have same-age peers; this will increase the odds of exposure to SHS^13^. In addition to that, the results of the study found that the prevalence and higher likelihood of male exposure to SHS may be explained by the higher prevalence of smoking among male adolescents in Malaysia and the tendency of males to mingle with other males; this might make non-smoking male adolescents more likely to be exposed to SHS from their smoking peers. We further hypothesize that female smokers tend to smoke in private, as female smoking is not a norm in Malaysian society. In addition, in single-parent families, the high prevalence of smoking and lack of parental supervision due to the demands of earning a living may consequently increase this group’s exposure to secondhand smoke (SHS). The finding may be explained by the high prevalence of smoking among non-married families and the busyness of earning a living, which cause parents not to be able to focus on their children and cause this group to be more at risk of being exposed to SHS^13,18^.

SHS exposure among ethnicities shows a trend that is almost the same as SHS exposure in 2017^13^, except among Chinese ethnic groups. The finding may be explained by the high prevalence of smoking among ethnic Malay adults in Bumiputra Sabah, Bumiputra Sarawak, and other. Still, the increase in ethnic groups may be explained by the interaction effect that shows a significant displacement of smoking among these ethnic groups. However, the interaction analysis shows that it affects all ethnic groups and sociodemographic status, such as gender, parents’ marital status, etc. Respondents who have one of their parents/guardians who smoke show higher odds of being more exposed to SHS compared to other factors in this study. The odds of exposure increased by almost two times compared to 2012. This further validates the hypothesis presented by Lim et al.^13^ and Ghazali et al.^17^ about the displacement of parents and household members smoking from public places to locations at home. This hypothesis is substantiated by the interaction analysis results, which show that the interaction between the independent variables (apart from the variable at least one parent smoked) shows that the group at risk is more likely to be exposed to SHS than the low-risk group (e.g. the interaction between gender and age group shows the likelihood for the low-risk group (13–15 years) is 3.7%, while the high-risk group (≥16 years) is 14%.

In contrast, the interaction between the independent variables (at least having parents who smoke) and other independent variables shows the opposite situation (e.g. parental smoking and current smokers show the difference in the low-risk group is 29.5% while the high-risk group is 17.5%). The finding indicates that low-risk groups have a higher probability of exposure to SHS if the respondent has at least one parent/guardian who smokes. The results showed the occurrence of displacement of smoking into the home from public areas. The findings are contradicted by the results of a systematic review and meta-analysis by Nanning et al.^28^ on SHS home exposure and Yao et al.^29^ among youth in the USA. However, this study’s results align with those of the Adda and Cognalia^30^ study in the USA, and those of the Ho et al.^31^ study among youth in Hong Kong. However, the findings of this study need to be substantiated by investigating the locality of exposure in future studies. In patriarchal societies such as Malaysia, smoking among the heads of the house in front of women and children is not prevented by other family members due to respect for the head of the family. These are common in LMICs; in addition to that, parents or guardians who smoke are more likely not to prohibit family members from smoking at home since they also smoke. Therefore, family members are free to smoke in the home area, which causes exposure to SHS among family members who do not smoke^13^. Furthermore, Lim et al.^32^, in a national population-based study, found that respondents who smoke or have family members who smoke are less likely to have a smoke-free house. Therefore, they allow visitors who visit the respondent’s house to smoke in the house, which may be a plausible factor contributing to this study’s findings.

### Limitations

The findings in this study are not without limitations. First, SHS exposure was measured verbally without biochemical verification. The cross-sectional design used in this study only measured the association between the dependent and independent variables, and no causality could be established. In addition, residual confounding might occur due to independent variables that are significant in other studies, such as the smoking status of household members^33^, home restriction of smoking^32,33^, smoking status of peers^33,34^, knowledge of SHS health hazards, and attitude towards smoking, which were not investigated^35^, and the age was classified as ‘young’ or ‘old’. The location of exposure to SHS was also not determined in this study. In addition, the findings from the current research can only be interpreted for secondary school-going adolescents rather than youth in other countries.

## CONCLUSIONS

The study demonstrated that exposure to SHS among school-going adolescents in Malaysia is still high; there is an indication of the displacement of smoking among parents from public places into the house. This result suggests that the policies and procedures were still unable to change the rate of SHS exposure. Health promotion targeted^35^ at ethnic Chinese parents and guardians not to smoke inside the house should be the main focus of an immediate intervention, in view of higher exposure to SHS among the low-risk youth in this ethnic group, and so reduce SHS exposure among the children. In addition, similar programs/interventions should include the parents/guardians from other ethnic groups. In addition, programs should be created in the interim to help parents and adolescents who smoke to quit by offering medication support or counselling.

## Supplementary Material



## Data Availability

The data supporting this research are available from the authors on reasonable request.

## References

[1] American Cancer Society, Health Risks of Secondhand Smoke; 2015. Accessed April 28, 2024. https://qa.cancer.org/content/dam/CRC/PDF/Public/6697.00.pdf

[2] US National Toxicology Program. 15th Report on Carcinogens. US Department of Health and Human Services. December 21, 2021. Accessed August 8, 2024. https://www.ncbi.nlm.nih.gov/books/NBK590769/

[3] Vital Strategies; Tobacconomics at Johns Hopkins University. The Tobacco Atlas: Secondhand smoke. Updated October 26, 2023. Accessed August 8, 2024. https://tobaccoatlas.org/challenges/secondhand-smoke/

[4] US Office on Smoking and Health. The Health Consequences of Involuntary Exposure to Tobacco Smoke: A Report of the Surgeon General. US Centers for Disease Control and Prevention; 2006. Accessed August 8, 2024. https://www.ncbi.nlm.nih.gov/books/NBK44324/20669524

[5] Ling J, Heffernan T. The cognitive deficits associated with second-hand smoking. Front Psychiatry. 2016;7:46. doi:10.3389/fpsyt.2016.00046 27047401 PMC4805605

[6] Safa F, Chaiton M, Mahmud I, Ahmed S, Chu A. The association between exposure to second-hand smoke and sleep disturbances: a systematic review and meta-analysis. Sleep Health. 2020;6(5):702-714. doi:10.1016/j.sleh.2020.03.008 32446663

[7] Intarut N, Pukdeesamai P. The prevalence of secondhand smoke exposure and related factors among schoolchildren in Northeast Thailand. F1000Res. 2020;9:1158. doi:10.12688/f1000research.26039.1 PMC7590888 33145013

[8] Yang X, Yan Z, Xu G, Tan Y, Zhu J. How secondhand smoke exposure affects tobacco use and smoking susceptibility of adolescents: sex and school differences. Tob Induc Dis. 2021;19(September):68. doi:10.18332/tid/140094 34539307 PMC8409096

[9] Ministry of Health Malaysia. National Strategic Plan for the Control of Tobacco & Smoking Products 2021-2030; 2021. Accessed April 28, 2024. https://www.moh.gov.my/moh/resources/Penerbitan/Rujukan/NCD/National%20Strategic%20Plan/NCDTembakau20212030.pdf

[10] World Health Organization. WHO report on the global tobacco epidemic, 2008: the MPOWER package; 2008. Accessed April 28, 2024. https://www.who.int/publications/i/item/9789241596282

[11] Government of Malaysia Food Act 1993: Control of tobacco product regulations. In Malay; 2004. Accessed April 28, 2024. http://www.tobaccocontrollaws.org/files/live/Malaysia/Malaysia%20-%20TC%20Regs%202004.pdf

[12] Melaka Bebas asap rokok. Website in Malay. Accessed April 28, 2024. https://mbar.moh.gov.my/2/

[13] Lim KH, Ghazali SM, Lim HL, et al. Prevalence and factors related to secondhand smoke exposure among secondary school-going adolescents in Malaysia: findings from Malaysia Global Health School Survey 2012 and 2017. Tob Induc Dis. 2021;19(June):50. doi:10.18332/tid/136029 34177412 PMC8204740

[14] Lim KH, Lim HL, Teh CH, et al. Is the implementation of smoke-free policies at workplaces associated with living in a smoke-free home? Findings from a national population-based study in Malaysia. Tob Induc Dis. 2019;17(June):51. doi:10.18332/tid/100692 31516494 PMC6662793

[15] Ministry of Health Malaysia. NHMS 2022 -Adolescent Health Survey- State Report. Updated 2023. Accessed August 8, 2024. https://iku.gov.my/nhms-ahs-2022-state-report

[16] Huang F, Zeng X, Di X, Xiao L, Liu S. Prevalence and determinants of secondhand smoke exposure among adolescent girls - China, 2019. China CDC Wkly. 2022;4(44):977-981. doi:10.46234/ccdcw2022.198 36483990 PMC9709297

[17] Ghazali SM, Huey TC, Cheong KC, et al. Prevalence and factors associated with secondhand smoke exposure among Malaysian adolescents. Tob Induc Dis. 2019;17(March):22. doi:10.18332/tid/102728 31582933 PMC6751970

[18] Ma C, Heiland EG, Li Z, Zhao M, Liang Y, Xi B. Global trends in the prevalence of secondhand smoke exposure among adolescents aged 12-16 years from 1999 to 2018: an analysis of repeated cross-sectional surveys. Lancet Glob Health. 2021;9(12):e1667-e1678. doi:10.1016/S2214-109X(21)00365-X 34571047

[19] Xi B, Liang Y, Liu Y, et al. Tobacco use and second-hand smoke exposure in young adolescents aged 12-15 years: data from 68 low-income and middle-income countries. Lancet Glob Health. 2016;4(11):e795-e805. doi:10.1016/S2214-109X(16)30187-5 27697484

[20] Kuwabara Y, Kinjo A, Kim H, et al. Secondhand smoke exposure and smoking prevalence among adolescents. JAMA Netw Open. 2023;6(10):e2338166. doi:10.1001/jamanetworkopen.2023.38166 37862017 PMC10589809

[21] Kuntz B, Lampert T. Trends in social inequalities in smoking and secondhand smoke exposure among adolescents in Germany: Benjamin Kuntz. European Journal of Public Health. 2015;25(suppl 3). doi:10.1093/eurpub/ckv170.019

[22] Gentzke AS, Wang TW, Marynak KL, Trivers KF, King BA. Exposure to secondhand smoke and secondhand e-cigarette aerosol among middle and high school students. Prev Chronic Dis. 2019;16:E42. doi:10.5888/pcd16.180531 30950787 PMC6464049

[23] Mohd Yusoff MF, Lim KH, Saminathan TA, et al. The pattern in prevalence and sociodemographic factors of smoking in Malaysia, 2011-2019: findings from national surveys. Tob Induc Dis. 2022;20(October):84. doi:10.18332/tid/152410 PMC9521184 36249344

[24] Lim KH, Amal NM, Hanjeet K, et al. Prevalence and factors related to smoking among secondary school students in Kota Tinggi District, Johor, Malaysia. Trop Biomed. 2006;23(1):75-84. 17041555

[25] Lagerweij NA, Kunst AE, Mélard N, et al. Where do teens smoke? Smoking locations of adolescents in Europe in relation to smoking bans in bars, schools and homes. Health Place. 2019;60:102213. doi:10.1016/j.healthplace.2019.102213 31585387

[26] McPherson M, Smith-Lovin L, Cook JM. Birds of a feather: homophily in social networks. Annual Review of Sociology. 2001;27:415–444. doi:10.1146/annurev.soc.27.1.415

[27] Lim KH, Teh CH, Nik Mohamed MH, et al. Exposure to tobacco secondhand smoke and its associated factors among non-smoking adults in smoking-restricted and non-restricted areas: findings from a nationwide study in Malaysia. BMJ Open. 2018;8(1):e017203. doi:10.1136/bmjopen-2017-017203 PMC5780697 29317411

[28] Nanninga S, Lhachimi SK, Bolte G. Impact of public smoking bans on children’s exposure to tobacco smoke at home: a systematic review and meta-analysis. BMC Public Health. 2018;18(1):749. doi:10.1186/s12889-018-5679-z 29925343 PMC6011268

[29] Yao T, Sung HY, Wang Y, Lightwood J, Max W. Sociodemographic differences among U.S. children and adults exposed to secondhand smoke at home: National Health Interview Surveys 2000 and 2010. Public Health Rep. 2016;131(2):357-366. doi:10.1177/003335491613100220 26957671 PMC4765985

[30] Adda J, Cornaglia F. The effect of bans and taxes on passive smoking. Am Econ J. 2010;2(1):1–32. doi:10.1257/app.2.1.1

[31] Ho SY, Wang MP, Lo WS, et al. Comprehensive smoke-free legislation and displacement of smoking into the homes of young children in Hong Kong. Tob Control. 2010;19(2):129-133. doi:10.1136/tc.2009.032003 20378586

[32] Lim KH, Lim HL, Kee CC, et al. Prevalence and factors associated with total smoking restriction at home in Malaysia: findings from a nationwide population-based study. Mal J Med Health Sci. 2019;15(3):20–28. Accessed August 8, 2024. https://medic.upm.edu.my/upload/dokumen/2019100108331804_MJMHS_0439.pdf

[33] Phetphum C, Noosorn N. Prevalence of secondhand smoke exposure at home and associated factors among middle school students in Northern Thailand. Tob Induc Dis. 2020;18(February):11. doi:10.18332/tid/117733 32165877 PMC7057047

[34] Alves RF, Precioso J, Becoña E. Smoking behavior and secondhand smoke exposure among university students in northern Portugal: relations with knowledge on tobacco use and attitudes toward smoking. Pulmonology. 2022;28(3):193-202. doi:10.1016/j.pulmoe.2020.03.004 32444313

[35] Junus S, Chew CC, Sugunan P, et al. Parental health risk perceptions and preventive measures related to children’s second-hand cigarette smoke exposure in Malaysia. BMC Public Health. 2021;21(1):1860. doi:10.1186/s12889-021-11825-2 34654405 PMC8518244

