# Supplementary material for: Exposure to secondhand smoke among school-going adolescents in Malaysia: Results from the National Health and Morbidity Survey (NHMS) 2022: Adolescent Health Survey (AHS)
Source: Tob Induc Dis. 2024 Oct 25;22:10.18332/tid/192002. doi: 10.18332/tid/192002 (PMC11504194; doi:10.18332/tid/192002)
Supplement: Supplementary file 1 [file TID-22-169-s1.pdf]

Supplementary Table 1. Probability for current e-cigarette use according to interactions between sociodemographic characteristics, lifestyle risk behaviours and parental factors

Sociodemographic characteristic or parental factor	Sociodemographic characteristic or parental factor	% (95% CI)
**Age (years)**	**Gender**	
13-15	Male	40.3 (38.3, 42.3)
16-18	Male	54.9 (52.1, 57.8)
13-15	Female	36.0 (34.4, 37.5)
16-18	Female	40.9 (39.1, 42.7)
**Age (years)**	**Ethnicity**	
13-15	Malay	37.2(35.3, 39.1)
16-18	Malay	51.1(48.8,53.4)
13-15	Chinese	44.8(41.4,48.2)
16-18	Chinese	40.9(37.2,44.8)
13-15	Indian	20.7(16.9,25.1)
16-18	Indian	20.7(16.5,25.5)
13-15	Bumiputra Sabah	40.7(33.9,47.8)
16-18	Bumiputra Sabah	51.0(44.9,57.1)
13-15	Bumiputra Sarawak	44.1(37.2,51.2)
16-18	Bumiputra Sarawak	57.2(51.0,63.2)
13-15	Others	41.8(36.4,47.4)
16-18	Others	51.2(44.2,58.2)
**Parental tobacco use**	**Gender**	
Yes	Male	64.7 (62.7, 66.5)
No	Male	32.0 (30.3, 33.8)
Yes	Female	60.6(58.7, 62.4)
No	Female	19.7(18.5, 20.9)

**Parental tobacco use**		
Yes	Current smoker	75.9 (73.5, 78.1)
No	Current smoker	51.2(48.2, 54.2)
Yes	Non-smoker	58.4 (56.7,60.2)
No	Non-smoker	21.9 (20.7, 23.2)
**Parental Tobacco Use**	**Marital status of parent/Guardian**	
Yes	Married	62.7 (61.4,64.2)
No	Married	25.3(24.1,26.5)
Yes	Separated/divorced/widow/widower	62.5 (59.8, 65.1)
No	Separated/divorced/widow/widower	31.1 (29.5,33.7)
**Sex**	**Ethnicity**	
Male	Malay	47.4(45.3, 49.6)
Female	Malay	37.1 (35.4,38.8))
Male	Chinese	42.1 (37.8, 46.5)
Female	Chinese	44.5 (41.3,47.7)
Male	Indian	23.0(18.5,28.2)
Female	Indian	18.6(14.9,23.0)
Male	Bumiputra Sabah	50.4(42.8,57.9)
Female	Bumiputra Sabah	40.8(35.6,46.2)
Male	Bumiputra Sarawak	55.8(49.4,62.1)
Female	Bumiputra Sarawak	42.3(37.0,47.8)
Male	Others	50.0(42.6,57.4)
Female	Others	40.3(34.5, 46.4)

**Parental Tobacco Use**	**Ethnicity**	
Yes	Malay	62.0 (60.1,63.8)
No	Malay	24.5(23.0,26.0)
Yes	Chinese	68.3 (64.7,71.7)
No	Chinese	30.4 (27.6, 33.2)
Yes	Indian	47.9(41.9,54.0)
No	Indian	13.8(10.7,017.6)
Yes	Bumiputra Sabah	61.8(55.8,67.4)
No	Bumiputra Sabah	31.6(27.2,36.4)
Yes	Bumiputra Sarawak	62.2(56.8,67.4)
No	Bumiputra Sarawak	36.9(31.6,42.4)
Yes	Others	64.7(56.7,72.0)
No	Others	29.8(24.7,35.4)

©2024 Lim K.H. et al.
